# Serum anti-neuraminidase antibody responses in human influenza A(H1N1)pdm09 virus infections

**DOI:** 10.1080/22221751.2019.1572433

**Published:** 2019-03-22

**Authors:** Herath M. T. K. Karunarathna, Ranawaka A. P. M. Perera, Vicky J. Fang, Hui-ling Yen, Benjamin John Cowling, Malik Peiris

**Affiliations:** a WHO Collaborating Centre for Infectious Disease Epidemiology and Control, School of Public Health, Li Ka Shing Faculty of Medicine, The University of Hong Kong, Hong Kong, Hong Kong SAR, People’s Republic of China; b Department of Veterinary Public Health and Pharmacology, Faculty of Veterinary Medicine and Animal Science, University of Peradeniya, Peradeniya, Sri Lanka

**Keywords:** Influenza, anti-neuraminidase antibody, protection, pandemic H1N1, seasonal H1N1, sero-diagnosis, cross-reactive antibody

## Abstract

Haemagglutination inhibition (HAI) antibody titres are a correlate of protection for influenza virus infection, but several studies have also demonstrated the protective role of anti-neuraminidase (anti-NA) antibodies. However, there is limited data on anti-NA antibody responses in naturally occurring human influenza. We investigated anti-NA antibody responses to pandemic N1 and seasonal N1 in 18 RT–PCR-confirmed patients with naturally acquired pandemic influenza A (H1N1) 2009 disease detected as part of a prospective community study of influenza. There were increases in neuraminidase inhibition (NAI) antibody titres to both pandemic and seasonal N1 antigens, with greater fold increases in those who had low levels of anti-pandemic N1 titres in acute sera. Of 18 patients with pandemic H1N1 infection, fourfold increases in antibody were observed by HAI in 11 (61%) patients, by anti-pandemic N1 inhibition in 13 (72%) or either in 15 of them (83%). Prior seasonal H1N1 virus infections had elicited cross-reactive anti-pandemic N1 antibody titres in some people prior to the emergence of the 2009 pandemic H1N1 virus. Antibody responses to the anti-N1 pandemic 2009 virus and cross-reactive responses to anti-seasonal N1 antibody were seen in influenza A pandemic 2009 infections. NAI antibodies can complement HAI antibody in sero-diagnosis and sero-epidemiology.

## Introduction

Influenza viruses pose global epidemic and pandemic threats [1]. Influenza A virus epidemics lead to infection of millions of humans worldwide every year [2], while pandemics, which occur at unpredictable intervals, cause millions of human deaths [3,4]. Annual influenza vaccination is the main strategy to prevent human influenza [5]. The effectiveness of the human influenza vaccine is primarily assessed by measuring serum anti-haemagglutinin (HA) antibody levels using the haemagglutination inhibition (HAI) assay and influenza vaccines are standardized by the amount of HA protein present in the vaccine formulation [6]. Both the United States Food and Drug Administration and the European Medicines Agency Committee for Medicinal Products for Human Use define HAI titres of ≥40 as the 50% “protective” titre for influenza virus infection [7].

However, several epidemiological studies and vaccine trials have demonstrated less than perfect correlation between HAI titres and protection in adults [8,9] as well as in children [10]. In particular, some individuals with high HAI titres can still be susceptible to infection, while other individuals with low HAI titres appear to be immune [8]. Recent data on seasonal influenza vaccination effectiveness have suggested that in some years vaccine protection was suboptimal, even though the vaccines met current seasonal vaccine standards and immunogenicity [11,12].

Seasonal influenza vaccines used before the 2009 pandemic (pdm) induced little or no cross-reactive antibodies against the novel pandemic influenza A H1N1 2009 (A(H1N1)pdm09) virus as detected using virus microneutralization assay [13]. Further it was observed that there was little or no seroconversion to pandemic A(H1N1)pdm09 influenza A virus following seasonal H1N1 vaccination in the animal models when measured by either HAI assay or microneutralization assay [14,15]. However, prior exposure to seasonal H1N1 influenza virus strains reduced weight loss, virus replication and transmission of influenza A(H1N1)pdm09 virus in experimentally infected mice, guinea pigs [14] and ferrets [15,16] and more importantly, limited morbidity to humans [17]. Considered overall, it was clear that exposure to the contemporary seasonal influenza viruses was unable to prevent A(H1N1)pdm09 virus infection, but did modulate morbidity in the absence of cross-reactive HAI antibodies.

On the contrary, numerous studies have pointed out the importance of neuraminidase (NA) protein in eliciting cross-reactive immunity against the influenza virus in animal models [18,19]. In humans, standard- dose or high-dose seasonal influenza vaccines have variable effect in eliciting serum anti-neuraminidase inhibition (NAI) antibodies against NA of pandemic H1N1 [18,20,21]. Human challenge studies conducted by Memoli and colleagues in 2016 demonstrated the importance of NAI titres as an immunological correlate of protection against influenza A(H1N1)pdm09 virus infection [12]. However, this study was carried out in healthy volunteers under experimental conditions and the relative importance of serum NAI antibody in conferring immunity against pandemic H1N1 2009 influenza A virus in natural infections has not been well studied [22,23].

In the current study we investigated pre- and post-infection NAI antibody titres to seasonal influenza A(H1N1) and pandemic influenza A(H1N1)pdm09 viruses in humans with naturally acquired influenza A(H1N1)pdm09 virus infection and correlated the protection afforded by cross-reactive anti-pdmN1 NAI antibody in baseline serum samples with clinical outcomes. Further we tested for cross-reactive anti-pdmN1 NAI antibody levels in serum samples collected in 2008, prior to the emergence of the 2009 H1N1 pandemic.

## Results

### Participants studied

We identified 18 symptomatic patients with RT–PCR-confirmed A(H1N1)pdm09 infection. The clinical and epidemiological details of these patients have been previously reported [24,25]. They ranged from 6 to 55 years of age (median age 30 years) and 9 of the patients were male. Of these, 12 were index cases identified at outpatient consultations, while 6 were household contacts of the index cases who acquired influenza virus infection, most likely from the index case. In all of the patients, the acute serum sample was collected ≤4 days after illness onset, and in five household contacts, at or prior to onset of disease (Table 1).

**Table 1.** NAI titres in patients with RT–PCR-confirmed pandemic influenza A H1N1 infection.

Patient (age/sex)	Date (dd-mm-yyyy) of positive RT–PCR result	Days relative to time of illness onset		NAI titres against pdmN1			NAI titres against seasonal N1			HAI titres against pandemic H1		
		Acute serum	Convalescent serum	Acute serum	Convalescent serum	Fold change	Acute serum	Convalescent serum	Fold change	Acute serum	Convalescent serum	Fold change
A (55/M)^a^	29-08-2010	3	34	320	320	1	40	80	2	<10	80	>8
B (36/F)	07-03-2011	0	28	80	320	4	40	160	4	<10	80	>8
C (28/F)	24-01-2013	−1	13	80	320	4	160	320	2	<10	20	>2
D (40/F)	31-01-2013	−2	23	160	160	1	160	320	2	<10	160	>16
E (47/F)^a^	29-01-2013	3	27	<10	320	>32	20	160	8	<10	<10	NA^b^
F (12/M)^a^	12-02-2013	4	21	<10	640	>64	20	1280	64	<10	80	>8
G (42/M)	12-02-2013	−1	16	<10	160	>16	10	80	8	<10	<10	NA
H (25/F)^a^	16-02-2013	4	33	<10	80	>8	320	1280	4	<10	320	>32
I (33/M)^a^	18-02-2013	3	20	10	80	8	20	40	2	40	320	8
J (24/F)^a^	26-02-2013	2	19	320	320	1	320	640	2	1280	1280	1
K (51/M)^a^	01-03-2013	3	23	320	160	−2	80	20	−4	<10	<10	NA
L (27/F)^a^	04-03-2013	4	21	<10	640	>64	320	2560	3	<10	160	>16
M (35/F)	13-03-2013	3	30	640	640	1	1280	640	−2	320	640	2
N (9/M)^a^	07-03-2013	3	21	<10	640	>64	20	640	32	<10	80	>8
O (30/M)^a^	16-03-2013	3	28	<10	80	>8	10	80	8	<10	<10	NA
*P* (6/M)^a^	25-03-2013	3	33	<10	80	>8	<10	<10	0	<10	40	>4
Q (30/F)	25-03-2013	0	30	20	2560	128	40	640	16	<10	1280	>128
R (30/M)	03-04-2013	3	30	20	640	32	80	640	4	<10	320	>32

^a^Index cases within family.

^b^NA: Not applicable because both acute and convalescent sera have titres <10.

### Anti-NA antibody response in RT–PCR-confirmed pandemic H1N1 infection

We measured anti-N1 NA antibody responses using two chimeric viruses that contained N1 of A/Hong Kong/415742/09 (pandemic H1N1) used for testing anti-pdmN1 antibody or A/Solomon Islands/03/06 (seasonal H1N1 vaccine strain in 2007/2008) used for testing for anti-seasonal N1 antibody with the HA (H6) gene from A/teal/Hong Kong/W312/97 and internal genes from A/Puerto Rico/8/34 (see methods). We observed a ≥4 fold increase in anti-pdmN1 NAI antibody titres in sera from 13 of the 18 RT–PCR patients (Table 1). All five who failed to demonstrate a four4fold increase in anti-pdmN1 antibody had high (≥160) baseline anti-pdmN1 titres in the acute serum. Two of them (patients J and M) also had high HAI titres in the acute serum, suggesting these may be re-infections, but the others had no detectable HAI titres, suggesting these were probably primary infections.

**Figure 1.**
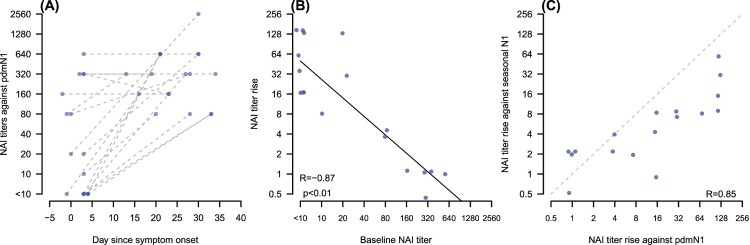
Panel A: Change of anti-pdmN1 NAI titres in relation to time after onset of illness in each patient. Panel B: Scatter plot of fold increase in anti-pdm N1 NAI titres to baseline NAI titre. Panel C: Scatter plot of the fold increase in NAI titres to pdmN1 versus seasonal N1.

All patients had the acute serum collected ≤4 days after onset of illness and five of them had the acute sample collected at or before onset of clinical symptoms. The changes of anti-pdmN1 NAI titres in relation to date after onset of illness at which the acute serum was collected are shown in Figure 1(A). Three patients who had anti-pdmN1 titres of ≥80 and HAI titres of <10 in the acute serum sample had these serum samples collected at or prior to onset of symptoms and thus these titres definitively represent cross-reactive antibody due to prior seasonal H1N1 infections. The fold increase in NAI titre was inversely correlated with the baseline NAI titre (*R* = 0.87; *p* < .01) (Figure 1(B)).

Twelve of the thirteen patients with a ≥4 fold increase in anti-pdmN1 antibody titre also had ≥4 fold increases in cross-reactive antibody to seasonal N1, some even when the baseline anti-seasonal N1 titres were as high as 320. The scatterplot of the fold increase in NAI titres to pdmN1 versus seasonal N1 is shown in Figure 1(C). The one person with a baseline anti-seasonal N1 NA titre of <10 (and also HAI titres of <10 to pdmN1) was a six-year-old child who did not demonstrate an antibody response to seasonal N1 although having an eightfold increase in titre to pandemic N1 and a fourfold increase in HAI titres.

In these 18 patients with RT–PCR-confirmed pdmH1N1 infection, a fourfold increase in HAI antibody was seen in 11 patients (61%), a fourfold increase in anti-pdmN1 NAI was seen in 13 (72%), while a fourfold increase in titres to either pdmH1N1 HAI or NAI was seen in 15 of them (83%) (Table 1). Fourfold increases in antibody titres in both NAI and HAI were seen in nine patients, while three had no antibody to either. It is notable that these three RT–PCR-confirmed patients who had no fourfold antibody increase all had high (≥320) baseline anti-pdmN1 NAI antibody titres. Two of them had high baseline HAI antibody titres (≥320) to A(H1N1)pdm09, suggesting that these may be re-infections with pdm09 virus (Table 1).

**Figure 2.**
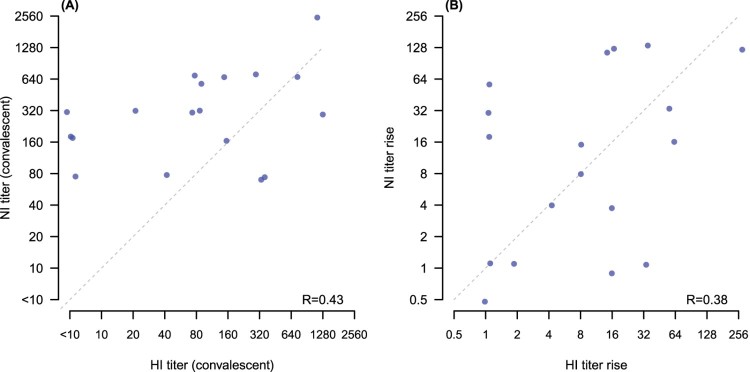
Correlation of NAI and HAI titres to A(H1N1)pdm09. Panel (A): correlation of convalescent HAI and NAI titres (*p* = .43). Panel (B): correlation of fold rises from acute to convalescence for HAI and NAI (*p* = .38). Diagonal dashed line in each panel shows the points of equal titres (A) or equal fold rises (B). Points have been jittered slightly to allow visibility of overlapping points.

We compared HAI and NAI antibody responses to A(H1N1)pdm09 and found no correlation either in convalescent titres (*p*-value .43) or in the fold rise from acute to convalescence (*p*-value .38) (Figure 2(A,B)).

### Correlation of baseline anti-pdmN1 NA titres with viral load or disease severity in patients infected with influenza A(H1N1)pdm09

**Figure 3.**
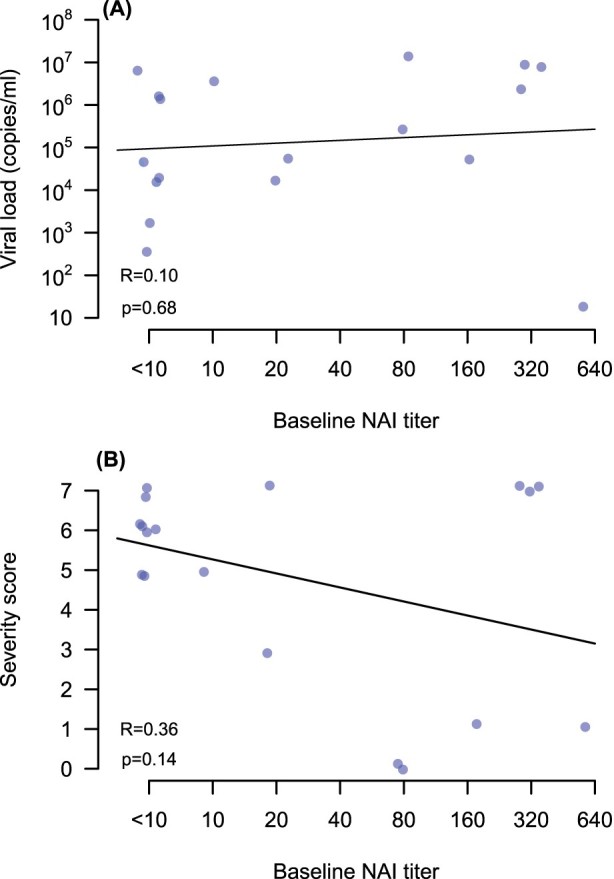
Correlation of A(H1N1)pdm09 NAI antibody titres in acute serum (*x* axis) with (A) viral load in nose and throat swabs and (B) disease severity scores in each patient.

There was no significant correlation between anti-pdmN1 antibody titres in baseline serum samples with viral load in nose/throat swabs (Figure 3(A)) or in disease severity scores (Figure 3(B)). The *P*-values for slope of linear regression lines fitted for the correlation between baseline anti-pdmN1 antibody titres and disease severity were 0.14. Since only two patients had detectable HAI titres to A(H1N1)pdm, the correlation between HAI titres and viral load or disease severity was not examined.

### Anti-NA antibody titres against pandemic N1 in human serum samples collected prior to 2009 (i.e. the emergence of the pandemic)

We tested anti-pdmN1 NAI antibody titres in 63 human sera (age from 7 to 74 years) collected in the year 2008, prior to the emergence of the pandemic H1N1 virus (Table 2). Twenty-nine (46%) of these individuals had no detectable anti-pdmN1 antibody (titre <10), 34 (54%) of them had anti-pdmN1 titres of ≥10 and 26 (41.3%) had titres of ≥20. Anti-pdmN1 titres of ≥10 were observed in 10 (35.7%) of 28 people aged less than 15 years and in 24 (68.6%) of 35 aged ≥15 years (Fishers exact test, two tailed, *p* = .012). Anti-pdmN1 titres of ≥20 were observed in 7 people (25%) aged less than 15 years and 19 (54.3%) aged ≥15 years (Fishers exact test, two tailed, *p* = .023).

**Table 2.** Anti-pdm N1 NAI antibody titres in sera collected prior to the emergence of the 2009 H1N1 pandemic.

Age (Years)	Date of serum collection	Anti-pdm N1 NAI titre	Anti-seasonal N1 NAI titre
7	05-12-2008	80	40
7	11-11-2008	<10	10
7	05-12-2008	<10	NA^a^
7	29-11-2008	10	NA
8	22-11-2008	<10	160
8	22-11-2008	<10	640
8	22-11-2008	<10	320
8	22-11-2008	<10	20
8	28-11-2008	<10	NA
8	12-12-2008	<10	NA
9	13-12-2008	<10	NA
10	22-11-2008	40	640
10	22-11-2008	20	640
10	19-12-2008	80	NA
10	29-11-2008	<10	NA
11	21-11-2008	80	2560
11	22-11-2008	10	80
11	21-11-2008	320	2560
11	21-11-2008	<10	40
11	06-12-2008	<10	NA
11	20-12-2008	<10	NA
12	13-12-2008	<10	NA
13	22-11-2008	<10	160
13	21-11-2008	640	1280
13	28-11-2008	10	NA
13	06-12-2008	<10	NA
14	29-11-2008	<10	80
14	19-12-2008	<10	NA
15	13-12-2008	20	NA
16	19-12-2008	20	NA
18	21-11-2008	<10	40
18	13-12-2008	<10	NA
19	21-11-2008	10	160
30	28-11-2008	20	NA
33	22-11-2008	40	1280
33	13-12-2008	20	NA
35	22-11-2008	20	160
37	22-11-2008	<10	NA
39	28-11-2008	<10	NA
40	06-12-2008	<10	NA
42	22-11-2008	<10	NA
43	22-11-2008	80	160
44	29-11-2008	20	NA
46	22-11-2008	<10	<10
46	22-11-2008	20	80
46	19-12-2008	<10	NA
47	21-11-2008	<10	<10
47	29-11-2008	<10	NA
48	21-11-2008	10	10
48	28-11-2008	<10	NA
51	21-11-2008	10	20
51	21-11-2008	320	640
51	22-11-2008	80	80
53	20-12-2008	20	NA
53	22-11-2008	10	40
54	20-12-2008	80	NA
57	13-12-2008	10	NA
57	06-12-2008	160	NA
65	22-11-2008	80	10
65	13-12-2008	20	NA
69	22-11-2008	20	20
74	13-12-2008	40	NA
74	13-12-2008	40	NA

^a^NA indicates serum not available for testing.

A subset of 30 of these sera was also tested for anti-seasonal N1 NAI antibody (Table 2). Twenty-eight of them had NAI antibody to seasonal N1, usually at higher titres than to pandemic N1. The two sera that did not have NAI antibody to seasonal N1 also had no antibody to pandemic N1.

## Discussion

Anti-NA antibody is increasingly being recognized as an independent correlate of protection in both human and animal influenza infection [18–20,23,26]. However, relatively little information is available on anti-NA antibody response and the effects of cross-reactive anti-NA antibodies during natural influenza A(H1N1)pdm09 infections in humans. In this study we demonstrated that prior seasonal influenza infections led to the development of an antibody that was cross-reactive with N1 of the A(H1N1)pdm09 virus in some persons prior to the emergence of the A(H1N1)pdm virus in 2009, with 34 (54%) of 63 sera tested having detectable antibody (≥10) to the N1 of A(H1N1)pdm09. In a subset of these sera tested for NAI antibody to seasonal N1, sera having NAI titres to pdmN1, all had NAI antibody to seasonal N1, usually to higher titre, compatible with the contention that anti-pdmN1 antibody detected in these sera was a result of the cross-reactivity of anti-seasonal N1 NAI antibody. Those younger than 15 years had a significantly lower prevalence (35.7%) of detectable anti-pdmN1 NAI antibody than those 15 years or older (68.6%). It is of interest that infection attack rates of H1N1pdm09 during the first wave of the pandemic in Hong Kong in persons aged 5–14 years, 15–19 years, 20–29 years, 30–39 years, 40–49 years and 50–59 years was 43.4 (95% CI 37.9–47.6), 15.8 (8.2–22.1), 11.8 (8.4–14.7), 4.3 (0.9–7.5), 4.6 (1–7.9) and 4 (1.1–7.5), respectively [27]. Thus, there was a marked decline in infection attack rates in those over 15 years of age, even though many of these persons had no detectable microneutralizing antibody to H1N1pdm09. Although other factors such as social mixing may have contributed to these age-specific differences in infection attack rates, it is possible that a pre-existing cross-reactive anti-pdmN1 antibody may have contributed to cross-protection.

Those who had A(H1N1)pdm09 infections consistently had increases in anti-pdmN1 antibody if their baseline pdmN1 antibody titre was low (≤20). Such individuals invariably had fourfold or greater responses to pandemic N1 and many of them also had fourfold or greater responses to seasonal N1. This may suggest that responses to pandemic N1 can be associated with cross-reactive seasonal N1 responses or that there is the equivalent of “original antigenic sin” where antibody to the previously generated seasonal N1 responses gets preferentially boosted by the pandemic N1 response [28]. It has been reported that the virus NA contains broadly reactive and cross-protective NAI epitopes that conserved between seasonal and pandemic N1 NA proteins [18]. The A/Solomon Islands/03/06 seasonal N1 we selected has remained antigenically conserved at least from 1991 up to 2006 and thus would capture anti-N1 responses elicited by seasonal H1N1 infections during this 15-year period. However, the seasonal A/Brisbane/59/2007 that circulated from 2007 to 2009 had undergone substantial antigenic drift [29].

Interestingly, in this cohort of 18 RT–PCR-confirmed A(H1N1)pdm09 infections, only 61% demonstrated a ≥4 fold increase in HAI antibody responses, but ≥4 fold NAI titre increases were seen in 72% and by either HAI or anti-N1 serology in 83%. This suggests that HAI tests underestimate influenza virus infections and that some of those patients may be identified by NAI testing. However, not all patients who had A(H1N1)pdm infections had NAI antibody titre increases to pandemic N1, especially those with high titres of cross-reactive baseline pandemic N1 antibody. But serologic testing by both HAI and NAI tests yields higher sensitivity in sero-diagnosis and sero-epidemiology. A recent study in New Zealand also found that a fraction of people followed in a longitudinal serologic study had ≥4 fold increases in NA titres but not HAI titres against influenza A(H3N2), although there was no RT–PCR confirmation in these cases [30,31].

Higher baseline anti-pdmN1 titres were not primarily related to collection of the acute serum later in the course of the illness. Because of the unusual study design where family contacts of index cases with influenza were recruited irrespective of symptoms, we had acute sera collected at or prior to the development of symptoms in five patients with A(H1N1)pdm09 infection.

An analysis to investigate a correlation between anti-pdmN1 in baseline serum samples with viral load or disease severity of these patients with A(H1N1)pdm09 infections did not identify a statistically significant clinical effect of higher anti-pdmN1 titres. However, our study was likely underpowered to demonstrate such an association. Our study did not directly investigate whether higher anti-N1pdm titres protected from A(H1N1)pdm infection. Associations between cross-reactive NAI antibody titres reducing the severity of previous pandemics have been reported [32–34].

An advantage of this study design was that the availability of sera from a family contact cohort allowed us to obtain sera at, or prior to, onset of symptoms in some patients. This stringent design was also responsible for the study limitation in regard to the small number of patients that could be studied.

It was recently reported that monoclonal antibodies to stalk region of the HA can lead to NAI activity presumably via steric hindrance of the NA enzymatic site [28]. It is not known how this relates to naturally elicited stalk-binding antibody responses that may occur following infections with pdm09H1N1 infection. Thus we cannot exclude the possibility that the NAI responses observed in our study may be contributed to by such antibody responses to the HA stalk. NA antibody responses assayed by ELISA assays to N1 may help clarify this situation, but how much cross-reactivity may exist in ELISA antibody assays to pdmN1 and seasonal N1 is not certain.

In conclusion, we found that prior seasonal influenza infections resulted in the presence of cross-reactive anti-pdmN1 NAI antibodies against A(H1N1)pdm09 influenza virus infection prior to the emergence of the 2009 pandemic. Those with low levels of anti-pdmN1 antibody in baseline serum showed the greatest fold increase in pdmN1 NA antibody. Antibody responses to pdmN1 were also associated with cross-reactive boost in anti-seasonal N1 antibodies. NAI antibody responses can complement HAI to increase sensitivity for sero-diagnosis and sero-epidemiology.

## Materials and methods

### Study participants and serum samples

We enrolled patients with medically attended influenza (“index cases”) along with their household contacts in a study of the transmissibility of influenza in households [24,25]. Patients were recruited in 2010, 2011 and 2013 which are second, and subsequent waves of pdm09 virus activity. Each index case had influenza confirmed by reverse transcription polymerase chain reaction (PCR) on a pooled nose and throat swab. Home visits were initiated within two days of index case enrolment for collection of pooled nose and throat swabs from household contacts, regardless of symptoms, for testing by PCR to identify secondary infections [25]. A subset of index cases and household contacts provided acute sera at the initial home visit and convalescent sera one month later. Disease severity scores were calculated for three groups of signs and symptoms – systemic signs and symptoms (measured body temperature ≥37.8°C, headache and myalgia), upper respiratory symptoms (sore throat and runny nose) and lower respiratory symptoms (cough and phlegm) [25]. The score for each patient was calculated by adding up the total number of signs and symptoms that were present.

A further 63 sera from persons ranging in age from 7 to 69 years were selected from a previous study on influenza vaccine efficacy in Hong Kong initiated in 2008, prior to the emergence of the 2009 pandemic [17]. Baseline pre-vaccination sera collected in 2008 were selected.

*Ethical approvals*: Written informed consent was obtained from all participants who were 18 years of age or older, and proxy written informed consent for participants under the age of 18 was obtained from parents or legal guardians. The study protocol was approved by the institutional review board at the University of Hong Kong.

### Cells, virus culture and NA antigens

Madin-Darby canine kidney (MDCK) cells were cultured in Dulbecco’s modified Eagle’s medium (DMEM) supplemented with 10% fetal bovine serum (FBS), 1% penicillin/streptomycin and 25 mM 4-(2-hydroxyethyl)-1-piperazineethanesulfonic acid (HEPES) at 37°C with 5% CO_2_. Two chimeric viruses were generated by reverse genetics as described previously [35] and contained the HA (H6) gene from A/teal/Hong Kong/W312/97, internal genes from A/Puerto Rico/8/34 and one of the following NA gene segments: N1 of A/Hong Kong/415742/09 (pandemic H1N1) used for testing anti-pdmN1 antibody or A/Solomon Islands/03/06 (seasonal H1N1 vaccine strain in 2007/2008) used for testing for anti-seasonal N1 antibody. The amino acid sequence of the NA of A/Hong Kong/415742/09 (H1N1) was identical to that of the prototype virus A/California/04/2009 (H1N1). The NA from A/Solomon Islands/03/06 was selected to represent seasonal N1 because of the antigenic stasis of the N1 protein among the seasonal H1N1 viruses isolated since 1991, despite the changes in the HA antigenicity [29]. The recombinant influenza viruses were rescued in 293 T cells and propagated in MDCK cells in culture medium supplemented with 1% penicillin, streptomycin and 1 μg/ml phenylalanyl chloromethyl ketone-treated trypsin (TPCK-trypsin).

### HAI assay

Serum specimens were tested with an HAI assay for antibody responses to the pandemic virus A/California/4/09 as part of a previous study performed in 2009–2010 [24].

### Enzyme-linked lectin assay (ELLA)

The inhibition of NA activity was measured using ELLA assays as described previously [36]. Briefly, the serum samples were heat-treated at 56°C for 45 min and then diluted serially (twofold) in 2-(*N*-Morpholino)ethanesulfonic acid (MES) buffer containing 1% BSA and 0.5% Tween 20 with the starting dilution of 1:10. Fifty microlitres from each dilution were added to duplicate wells in plates coated with fetuin (Sigma-Aldrich, Hong Kong). An equal volume (50 μl) of selected virus dilution was added to all serum-containing wells. Eight wells in the first row of the 96 well plate contained only MES – bovine serum albumin (BSA) buffer (no serum) and served as virus (antigen) positive control, while eight wells in the 12th row of the same plate served as the background control (without virus/antigen). These plates were subsequently sealed and incubated for 18 h at 37°C in a humidified incubator.

After incubation, plates were washed six times with wash buffer and 100 μl of an optimized dilution (1:1000) of horseradish peroxidase-conjugated peanut agglutinin lectin (PNA-HRPO, Sigma-Aldrich) in MES buffer containing 1% BSA was added to each well. The plates were incubated for 2 h at room temperature and washed three times before the O-phenylenediamine dihydrochloride (OPD, Sigma-Aldrich) substrate, prepared according to the manufacturer’s instructions, was added. The colour reaction was stopped after 10 min by adding 1N H_2_SO_4_. The plates were read at wavelength 492 nm for 0.1 s using FLUOstar OPTIMA (BMG Labtech) 96 well plate reader. The NAI titres were defined as the reciprocal of the last dilution that resulted in at least 50% inhibition in comparison with control. Our laboratory had previously participated in the CONSISE standardization of ELLA tests across laboratories [37,38].
